# Projected local rain events due to climate change and the impacts on waterborne diseases in Vancouver, British Columbia, Canada

**DOI:** 10.1186/s12940-019-0550-y

**Published:** 2019-12-30

**Authors:** Bimal K. Chhetri, Eleni Galanis, Stephen Sobie, Jordan Brubacher, Robert Balshaw, Michael Otterstatter, Sunny Mak, Marcus Lem, Mark Lysyshyn, Trevor Murdock, Manon Fleury, Kirsten Zickfeld, Mark Zubel, Len Clarkson, Tim K. Takaro

**Affiliations:** 1 Faculty of Health Sciences, Simon Fraser University, 8888 University Dr. BLU 11300, Burnaby, British Columbia Canada; 2 British Columbia Centre for Disease Control, Vancouver, British Columbia Canada; 3 School of Population and Public Health, University of British Columbia, Vancouver, British Columbia Canada; 4 Pacific Climate Impacts Consortium, University of Victoria, Victoria, British Columbia Canada; 5 George and Fay Yee Centre for Healthcare Innovation, University of Manitoba, Winnipeg, Manitoba Canada; 6 Vancouver Coastal Health, Vancouver, British Columbia Canada; 7 Department of Geography, Simon Fraser University, Burnaby, British Columbia Canada

**Keywords:** Waterborne disease, Climate change, Extreme precipitation, Downscaled climate projections, Future health impact

## Abstract

**Background:**

Climate change is increasing the number and intensity of extreme weather events in many parts of the world. Precipitation extremes have been linked to both outbreaks and sporadic cases of waterborne illness. We have previously shown a link between heavy rain and turbidity to population-level risk of sporadic cryptosporidiosis and giardiasis in a major Canadian urban population. The risk increased with 30 or more dry days in the 60 days preceding the week of extreme rain. The goal of this study was to investigate the change in cryptosporidiosis and giardiasis risk due to climate change, primarily change in extreme precipitation.

**Methods:**

Cases of cryptosporidiosis and giardiasis were extracted from a reportable disease system (1997–2009). We used distributed lag non-linear Poisson regression models and projections of the exposure-outcome relationship to estimate future illness (2020–2099). The climate projections are derived from twelve statistically downscaled regional climate models. Relative Concentration Pathway 8.5 was used to project precipitation derived from daily gridded weather observation data (~ 6 × 10 km resolution) covering the central of three adjacent watersheds serving metropolitan Vancouver for the 2020s, 2040s, 2060s and 2080s.

**Results:**

Precipitation is predicted to steadily increase in these watersheds during the wet season (Oct. -Mar.) and decrease in other parts of the year up through the 2080s. More weeks with extreme rain (>90th percentile) are expected. These weeks are predicted to increase the annual rates of cryptosporidiosis and giardiasis by approximately 16% by the 2080s corresponding to an increase of 55–136 additional cases per year depending upon the climate model used. The predicted increase in the number of waterborne illness cases are during the wet months. The range in future projections compared to historical monthly case counts typically differed by 10–20% across climate models but the direction of change was consistent for all models.

**Discussion:**

If new water filtration measures had not been implemented in our study area in 2010–2015, the risk of cryptosporidiosis and giardiasis would have been expected to increase with climate change, particularly precipitation changes. In addition to the predicted increase in the frequency and intensity of extreme precipitation events, the frequency and length of wet and dry spells could also affect the risk of waterborne diseases as we observed in the historical period. These findings add to the growing evidence regarding the need to prepare water systems to manage and become resilient to climate change-related health risks.

## Introduction

Climate change is expected to cause a global rise in temperature and sea level, as well as changes in the frequency and intensity of precipitation [1, 2]. Climate change is likely to have a negative impact on human health overall, at both population and individual levels, by exacerbating prevalent conditions like respiratory and cardiovascular disease as well as an expansion of emerging infectious diseases [3]. In particular, climate change is expected to increase the global burden of waterborne acute gastrointestinal infections (AGI) [4, 5] primarily due to an increase in the frequency and intensity of extreme precipitation events [2, 6].

Several studies have reported that two important AGI, cryptosporidiosis and giardiasis, have seasonal variability and may therefore be affected by climate change [7–10]. Extreme precipitation events have been implicated in several waterborne AGI outbreaks [11–14] and in sporadic AGI [15]. Extreme precipitation may increase pathogen transfer from environmental reservoirs (e.g. animal manure) into surface water either directly, by increasing stream discharge, which increases turbidity and promotes the re-suspension of infectious cysts/oocysts from river sediments [16], or indirectly, by increasing overland runoff into water systems [17–19]. Such increases in water turbidity can reduce the efficacy of drinking water treatment [4, 20, 21]. Ascertaining the vulnerability of drinking water systems to extreme weather events in the present and the future is necessary for climate change adaptation approaches to protect public health.

In a related previous study [15], we investigated the relationship between extreme precipitation events (defined as 7-day cumulative weekly precipitation exceeding the 90th percentile in the historical record), drinking water turbidity and cryptosporidiosis and giardiasis for an urban surface drinking water system (DWS) in Metro Vancouver, British Columbia, Canada. The study found that extreme precipitation led to significantly increased turbidity, and cryptosporidiosis and giardiasis risk and the risk was greater for precipitation following a dry period [15]. After the previous study period (1997–2009) this DWS installed filtration for two of its three surface water sources to increase the protection from both direct and indirect turbidity-causing events. While the previous study provided historical information about the relationships between extreme precipitation and disease, the objective of our current study is to investigate the change in cryptosporidiosis and giardiasis risk due to climate change, primarily change in extreme precipitation, had these filtration systems not been installed.

## Methods

Estimation of the future impact of climate change on the risk of cryptosporidiosis and giardiasis required two stages:1) Characterization of the exposure-outcome relationship between precipitation and disease through analysis of historical data (1997–2009) using distributed lag non-linear Poisson regression models; and 2) projection of the exposure-outcome relationship to future periods (2020–2099) using climate model predictions to derive expected annual numbers of cases under various climate change scenarios. The details of Stage 1 were described previously [15].

### Stage 2 – future impact of climate change on AGI

#### Model

The final model from Stage 1 was used to predict weekly case counts of cryptosporidiosis and giardiasis between 2020 and 2099 by incorporating corresponding predictions of future values of the explanatory variables. The case counts were calculated using the following log-linear model,

1 $$ E\left({Y}_t\right)=\exp \left(a+s\left({\upsilon}_t| df\right)+{\gamma}_1\sin \left(2\cdot \pi \cdot \frac{t}{52}\right)+{\gamma}_2\cos \left(2\cdot \pi \cdot \frac{t}{52}\right)+{I}_1\cdot g\left({w}_{t:t-8}| lag\; df,\mathit{\operatorname{var}}\; df\right)+{\gamma}_3H{W}_t+{\gamma}_4 PO{P}_t\right) $$

where, *t* represents current week, E(Y_t_) is the expected number of cases per week (or week *t, s* is a cubic spline that adjusts for secular trends with variable *v* representing ordered discrete count of weeks in the data. The flexibility parameter in *s* was set to 7 degrees of freedom (df) per year. γ_1_and γ_2_ represent coefficients of the harmonic terms to control for season. *G(w)* is a two-dimensional function and defines the distributed lag nonlinear predictor of *w*, the weekly precipitation for lags 0–8, and is controlled by independent parameters across its values and across its lags (*var df*, and *lag df*). I_1_ is an indicator variable representing preceding dry period equal to 1, if there were less than 30 days with at least 0.1 mm/day precipitation in the preceding two months and equal to 0 otherwise. HW is another indicator variable indicating a week with national holiday to account for differences in reporting and access to health care during such weeks, POP_*t*_ captures population growth over time and represents the logarithm of the provincial population at time *t.*

#### Data

Daily precipitation projections (mm/day) for Representative Concentration Pathway (RCP) 8.5 for January 5, 2020 to December 26, 2099 were obtained online from the Pacific Climate Impacts Consortium (PCIC) data portal [22]. Twelve sets of projections were obtained. PCIC derives these projections by statistical downscaling of 12 global climate models (GCMs) (Table 1) from the Coupled Model Inter-comparison Project Phase 5 [23]. The downscaled projections were generated using monthly bias-correction and spatial disaggregation (BCSD), a methodology commonly used in hydrologic modeling [24]. RCPs represent a range of greenhouse gas (GHG) concentration scenarios up to 2100, based on assumptions about economic activity, energy sources, population growth and other socio-economic factors. RCP 8.5 is a ‘status quo’ scenario characterized by increasing GHG emissions over time leading to high GHG concentration levels up to the year 2100 [25]. The precipitation projections for RCP 8.5 entered Eq. (1) as projected mean weekly precipitation (*w*_*t*_) at the grid corresponding to the geographic coordinates of N49.44, W-122.97 decimal degrees (i.e. the corresponding grid point near the Seymour Dam used in our historical precipitation data). The expected cases derived from model (1) were aggregated to annual counts and compared to historical annual counts as the percent change (from historical) in annual incidence of disease during the 2020s (2020–2039), 2040s (2040–2059), 2060s (2060–2079), and 2080s (2080–2099). The results are presented using the ensemble mean of all 12 projections along with the minimum and maximum to represent natural climate variability and uncertainty across climate models.

**Table 1:**
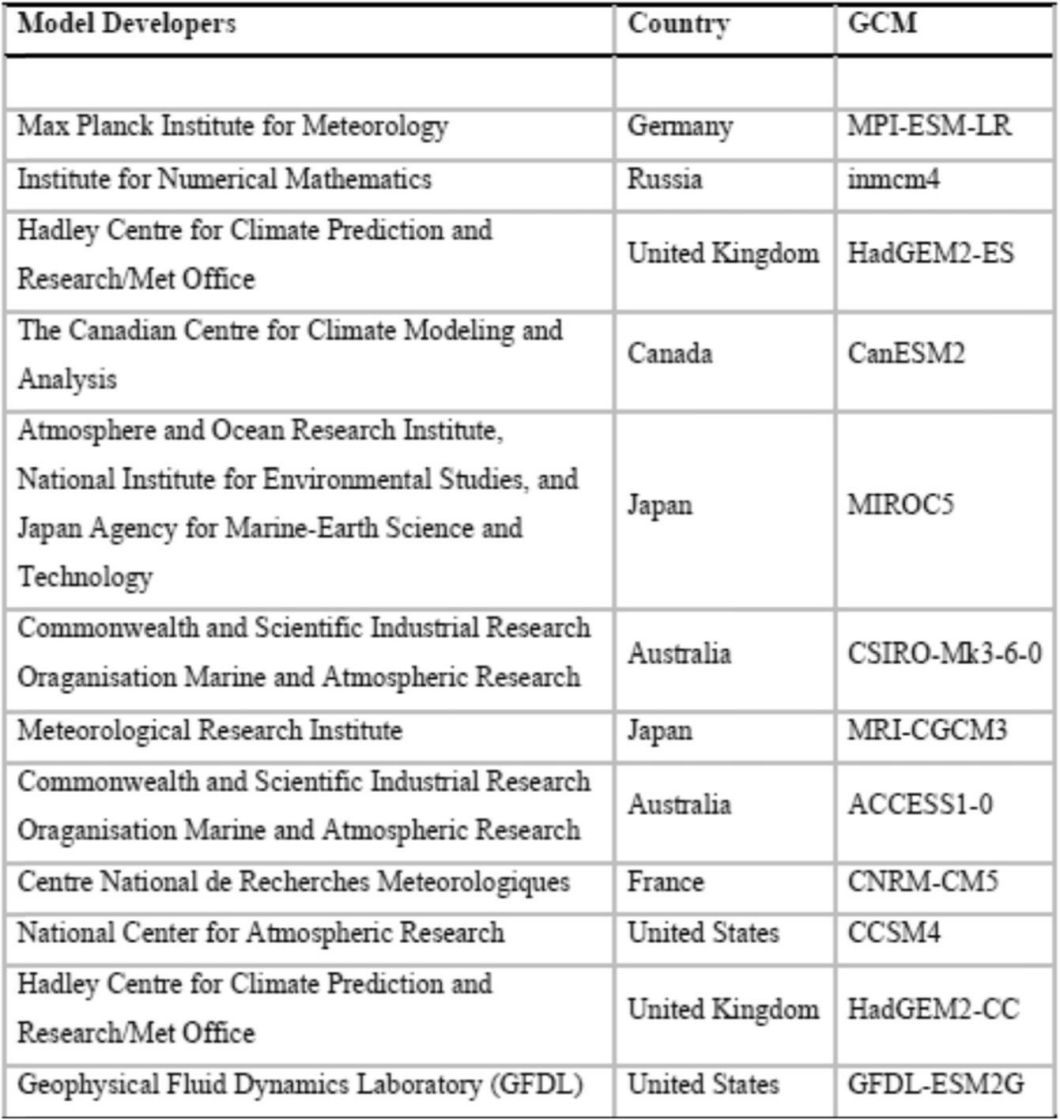
The climate modeling institutions and corresponding global climate models (GCMs) used for downscaled precipitation projections by PCIC

Daily temperature and precipitation data for the Seymour reservoir watershed and modelled future projections were provided by the Pacific Climate Impacts Consortium (PCIC). The historical data are derived from daily gridded weather observation data [26] and are spatially interpolated at a 1/12 degree (~ 6 × 10 km) resolution. We used interpolated data for the historical period so that it would be comparable to the PCIC statistically downscaled future climate simulations at the same resolution.

All data management and statistical analysis tasks were carried out using the statistical software R distributed-lag-non-linear model package version 2.0.6 [27].

## Results

**Fig. 1:**
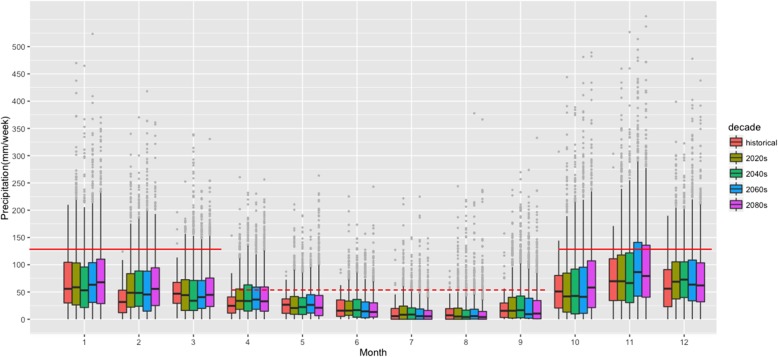
Projected weekly precipitation by month from the 2020s to the 2080s across an ensemble of 12 climate models. Solid red and dashed red represents historical 90th percentile precipitation from rainy and dry seasons respectively. The upper whisker of the box plot (solid vertical line) extends from the hinge to the highest value that is within 1.5 * IQR of the hinge, where IQR is the inter-quartile range. Single points are values above 1.5 * IQR

**Fig. 2:**
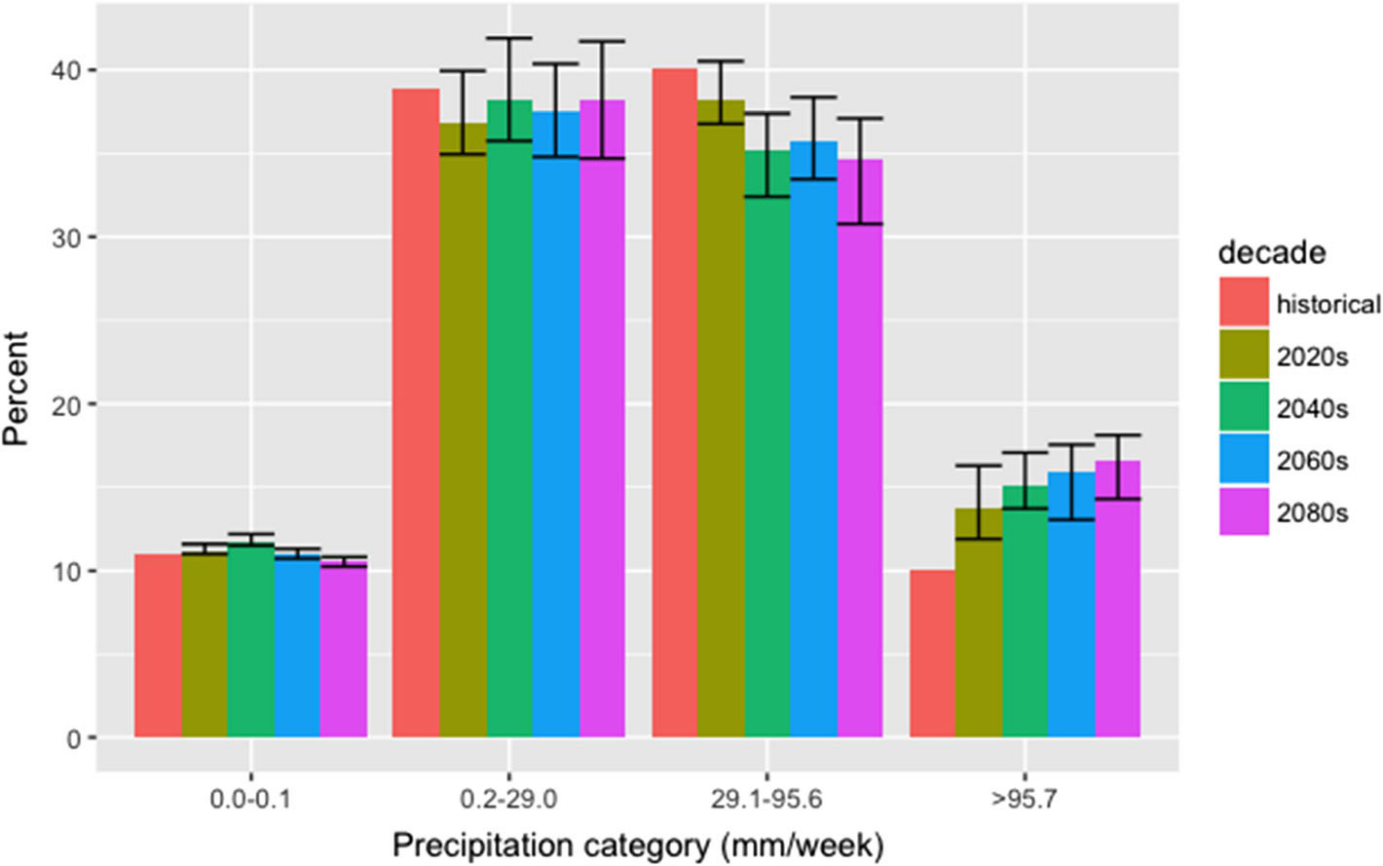
The percentage of weeks with observed or projected precipitation corresponding to various categories in historical and future time periods (2020s, 2040s, 2060s, and 2080s). Bars represent the mean of 12 climate models and the error bars represent minimum and maximum percentage change expected across 12 climate models

For the central watershed in the study area of metropolitan Vancouver (Seymour Reservoir, see map Additional file 1: Figure S1), mean weekly precipitation is projected to generally increase in the wet season (October to March) and decrease in the dry season (April to September) in the 2080s compared to the historical period (Fig. 1). Further, the proportion of weeks with small amounts of precipitation (0–29 mm) are projected to remain stable, those with moderate precipitation (29-96 mm) are projected to decrease and weeks above the 90th percentile of historical (> 96 mm/week) show an increase in the future (Fig. 2).

In the absence of the additional water filtration which was installed, the incidence of cryptosporidiosis and giardiasis was projected to increase in the future in association with the projected changes in precipitation (Table 2). Across the twelve models, the combined incidence relative to 1970–2000 mean baseline increased by an average of 6% in the 2020s, 8% in the 2040s, 12% in the 2060s and 16% in the 2080s. Compared to the historical average of 558 cases per year [15], this corresponds to an expected 591 cases per year in the 2020s (ensemble range: 559–627) to 649 cases per year in the 2080s (ensemble range: 613 to 694). Differences in magnitude of climate projections between models occur due to both natural climate variability and model construction and uncertainty; each model simulation can represent different “phases” of climate variability which can be larger than the structural difference between models. Some climate model projections consistently resulted in either higher or lower changes in disease incidence. In our AGI model the projected case count increases ranged from 10 to 24% by the 2080’s relative to the historical period. To reduce the influence of these potential outliers in individual runs we removed the upper and lower 10% of model values at each year before averaging the remaining eight projections to arrive at our final annual estimate.

**Table 2:**
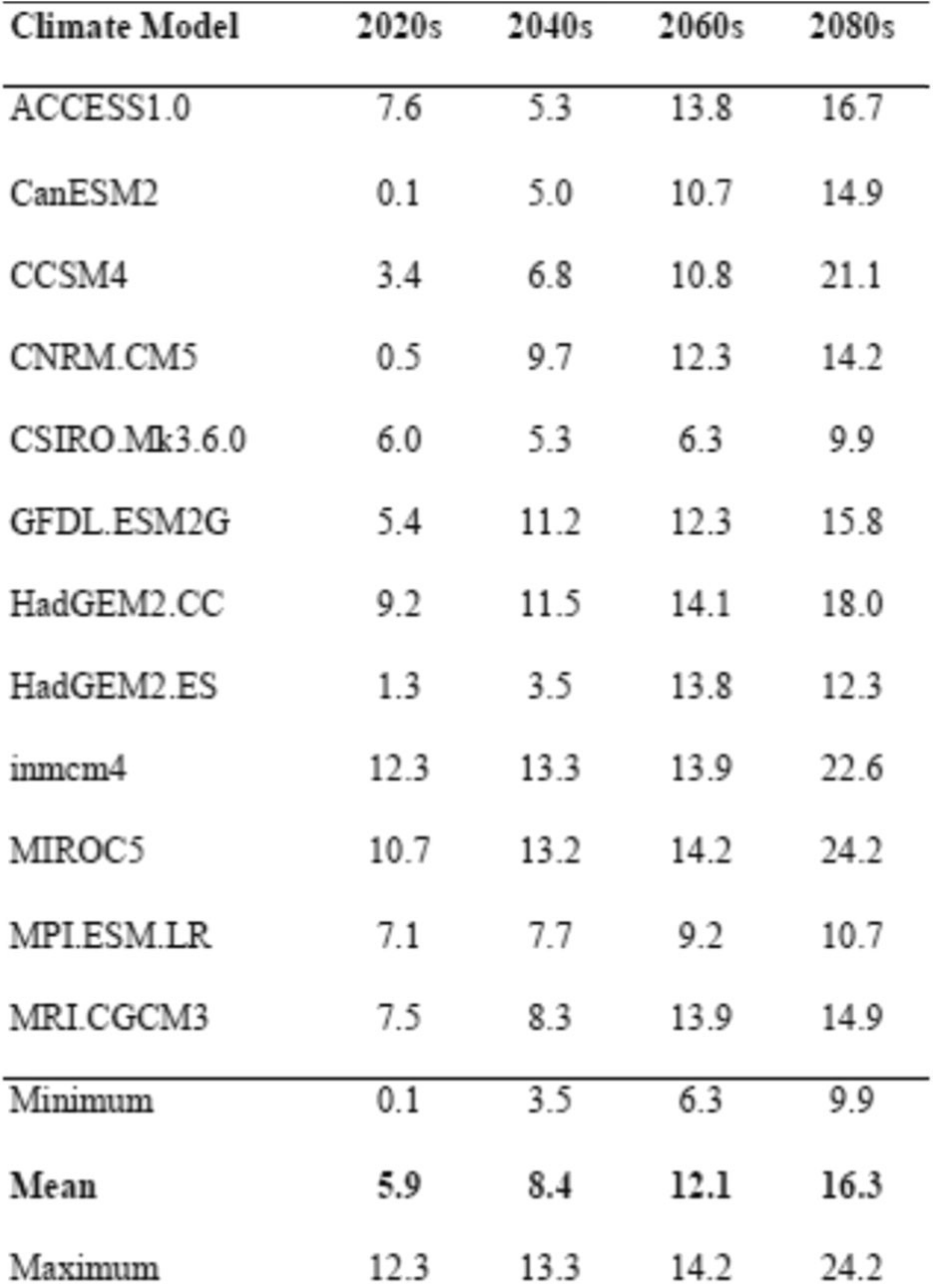
Percentage change in annual cases of cryptosporidiosis and giardiasis in the 2020s, 2040s, 2060s and 2080s compared to 1997–2009 based on projected precipitation from different climate models

**Fig. 3:**
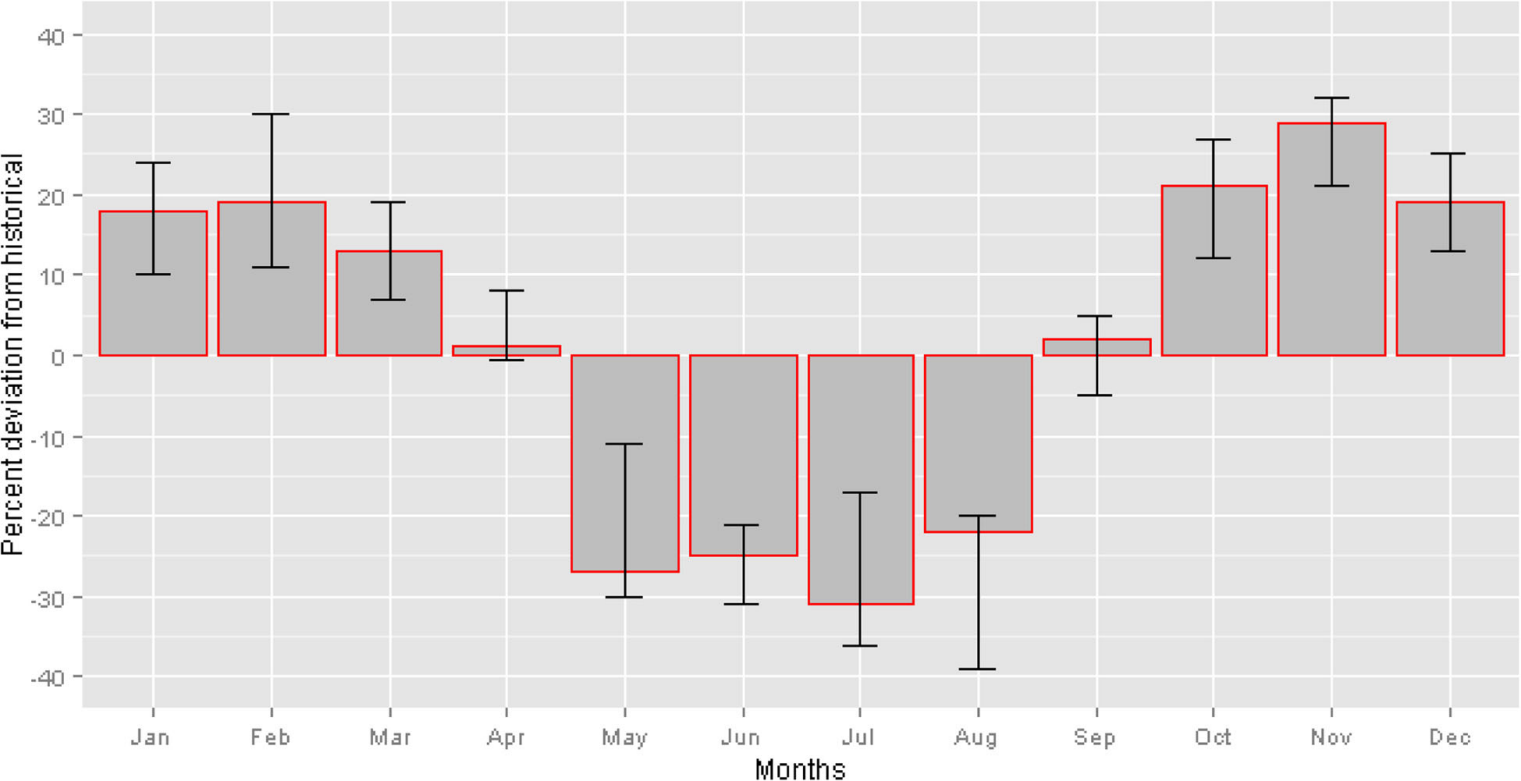
Mean percent change in monthly cases of cryptosporidiosis and giardiasis morbidity in 2080s as compared to historical (1997–2009) across 12 climate models. The error bars represent the maximum and minimum percent change across the 12 climate model ensemble

Examining the AGI model results by month reveals the incidence of cryptosporidiosis and giardiasis is expected to decrease in May to August by up to 31% compared to the historical baseline, but increase by up to 29% in October to March (Fig. 3). The range in future projections compared to historical monthly case counts typically differed by 10 to 20% across climate models but the direction of change was consistent regardless of the model.

The retrospective model validation indicated a reasonable model fit with mean absolute error of ~ 1 case per week. Diagnostic plots showed no gross indicators of model misspecification (Additional file 1). Using the observed Environment Canada (EC) weather station data as the ‘gold standard’ for weekly precipitation, we compared the weekly cumulative precipitation for EC with the PCIC interpolated precipitation for the grid containing the EC weather station. This generated a specificity (true negative/(false positive + true negative)) for PCIC data to detect observed extremes of precipitation of 97% while the sensitivity was 75% ((true positive/(true positive + false negative)).

## Discussion

Ours is the first study to assess the impact of future precipitation on cryptosporidiosis and giardiasis utilizing projections from a broad range of downscaled global climate models. It provides evidence that these waterborne illnesses can be impacted by climate change. We first evaluated the climate-disease relationship at a municipal water system scale to assess the epidemiologic relationship between precipitation and the risk of waterborne pathogens. Having identified extreme precipitation as the primary influence, we then used downscaled precipitation projections to assess the impact of future climate on disease [28, 44]. This approach identifies future impacts that are most relevant to the study area and is based on exposure-outcome relationships observed in this same area [15].

The results from this study indicate that extreme precipitation will become more common in the future in Metro Vancouver. This is consistent with the Intergovernmental Panel on Climate Change (IPCC) Special Report on Extremes for the region [29] and BC government projections [30]. Since these extreme weather events contribute to water contamination through environmental factors such as increased turbidity, effective adaptation strategies that include turbidity control with filtration are needed to reduce water system vulnerability. Many medium to small-scale drinking water systems are operated with limited resources and are often unfiltered with poor infrastructure. These may be particularly at risk from extreme weather events [31, 32]. If water treatment mitigation measures had not been implemented for Metro Vancouver as they were between 2010 and 2015, the incidence of cryptosporidiosis and giardiasis would likely increase overall between the 1990s to the 2080s driven primarily by expected changes in precipitation and increase in the population-attributable-risk. Within this overall annual increase, more cases would be expected from October to March and fewer would be expected from May to August [33]. Since 2009 the DWS for Metro Vancouver has had staged increases in the proportion of filtered finished water beginning with the Seymour Reservoir, followed by the connection of the Capilano Reservoir water to Seymour Capiliano filtration plant in 2014. The Coquitlam Reservoir, the third source for the system, is unfiltered relying upon ozonation as pre-treatment, UV (added in 2014 to enhance treatment), chlorine and pH control for treatment. Though it varies, usually about half of Metro Vancouver’s finished water is filtered [45]. We would expect these interventions have reduced the effect size seen between 1997 and 2009, but because turbidity remains a feature of source water from surface sources, we would expect the relationship between extreme rain events and waterborne disease risk to remain.

Previous studies have reported on the impacts of climate change on diarrheal morbidity and mortality [34–36]. These studies suggest variable increases in diarrheal disease arising from temperature change based on large-scale GCMs. For example, one study projects a 22 to 29% increase in risk of diarrhea by 2070–2099 in six study regions of the world (excluding North America) compared to 1961–1990, based on projected changes in temperature [35]. The World Health Organization (WHO) estimates a 5% increase in diarrheal morbidity for each 1 degree Celsius increase in temperature [37]. A study from Lebanon found an increased burden of food and waterborne illnesses under future scenarios of intensive industrial development and projected changes in temperature [34]. It is difficult to generalize these results to other contexts such as our region in western Canada. First, in previous work, future risks of waterborne illness are extrapolated from studies investigating several pathogens at once, some of which are primarily foodborne. Second, those studies encompass large spatial scales, e.g., national or continental, while factors influencing waterborne disease risk likely act at much smaller scales e.g. watersheds or supply areas. Finally, previous work has not accounted for projected changes in the intensity and frequency of future precipitation, a well-known environmental predictor of water borne illness [11–13, 30].

Our final model, built on historical data, did not include temperature since this was not a significant factor controlling cryptosporidiosis and giardiasis in the historical period [15]. While temperature has consistently been associated with bacterial acute gastrointestinal illness [38, 39], such a link with cryptosporidiosis and giardiasis is less clear [40]. The model does however account for seasonal variation by including month as a factor in the model which acts as a proxy for temperature. Summer drought may increase the risk of waterborne diseases due to concentration of pathogens that are then washed into a DWS [5], a phenomenon also observed in the lagged response of disease to precipitation in the present study. In the future very high temperatures and the risk of drought in the summer may lead to a muted summer decrease compared with the model we developed.

The estimated additional number of waterborne disease cases reported in the future is relatively small (approximately 91 more cases per year). However, it is estimated that less than 3% of such cases are currently reported to public health authorities (1 out of 48.5 and 40.7 cases of cryptosporidiosis and giardiasis, respectively) [41]. If this holds for our projections, then between 3703 to 4414 additional cases per year could be attributed to climate change. Additionally, for the precipitation estimates sensitivity is less than specificity, so our disease estimates likely represent a lower-bound since we are necessarily conservative in the estimate of extreme rain events.

In addition to the predicted increase in the frequency and intensity of extreme precipitation events, the frequency and length of wet and dry spells could also affect the risk of waterborne diseases as we observed in the historical period. We have less confidence in the modeling for the future number of dry to wet cycles per annum due to the design of the downscaling method. This problem is described in detail by Cannon, et al. [42] Analysis of several common indicators of climate extremes near the study area project changes to wet and dry spell lengths [43, 33].

The estimation of the future burden of waterborne illness presents considerable challenges. Several factors that may influence how climate change will affect disease risk can be difficult to model due to the lack of data and our limited understanding of future biological (host-agent-environment) interactions. These include changes in drinking water system characteristics (e.g., improvement in water quality and infrastructure), increased adaptation efforts to climate change (e.g., better watershed management, health services) or changes in behavior influencing the risk of disease (e.g., change in the proportion of the population drinking tap water). Additionally, the long-term illness projections (2020–2080) are based upon a relatively short historical period (1997–2009). Our findings need confirmation in other populations using unfiltered surface water systems with longer historical periods. Examining the range of possible outcomes could assist municipalities in prioritizing building resilience to extreme rain events into their water systems.

In this study, we developed a modeling framework that can be used with available downscaled precipitation projections to provide empirical evidence of how precipitation is likely to change and affect the incidence of cryptosporidiosis and giardiasis in the absence of sophisticated filtration. Given that downscaled future climate projections are readily available for Canada from PCIC, Ouranos (Quebec), USA (Scripps Institute) and other jurisdictions, a modeling framework such as ours can help understand the risk future climate poses to health through drinking water systems such that possible mitigation strategies can be implemented. We have designed an open-access software tool named ImpactR for the Public Health Agency of Canada to enable such estimation for those with access to local disease registries. ImpactR, enables calculation of future waterborne illness disease rates, then using 1/12 degree (~ 6 X 10 km) precipitation projections from PCIC to estimate future rates. Model coefficients and covariates selected in Stage 1 may require new calibration should the model be applied in climate regimes dissimilar to Metro Vancouver to account for contextual variations in local precipitation, case counts, and turbidity. The tool is available here: http://www.bccdc.ca/health-info/disease-types/foodborne-waterborne-diseases.

## Conclusion

Without the filtration added in 2010–2015, the risk of cryptosporidiosis and giardiasis would have been expected to increase with climate change, particularly precipitation changes, in our study area. These findings add to the growing evidence regarding the need to prepare, manage and become resilient to climate change-related risks. Although uncertainties remain in modelling the host-pathogen-interactions, we present a framework to study the future impact of water-borne disease illness using downscaled climate data that is suitable for local conditions. Further research is necessary to incorporate multiple exposure pathways, health outcomes and water system specific drivers to understand the present and future waterborne risks more clearly.

## Supplementary information

**Additional file 1: Figure S1.** Map depicting the watersheds and supply areas for the Metro Vancouver Drinking Water System (Source: City of Surrey, BC, Canada, 2015). **Figure S2**. Comparison of weekly cumulative precipitation observed at Environment Canada weather station 698 and Pacific Climate Impacts Consortium (PCIC) interpolated precipitation for the grid containing the weather station (1997–2009). **Figure S3.** Time series of weekly cases of cryptosporidiosis and giardiasis, weekly cumulative precipitation, preceding dry days and average weekly turbidity from 1997 to 2009.

## Data Availability

The data will be available as permitted under the SFU Research ethics, B.C. Centre for Disease Control and Ministry of Health data-use agreement and made available on the following website: http://www.bccdc.ca/health-info/disease-types/foodborne-waterborne-diseases.

## Abbreviations

AGI: Acute gastrointestinal infections
BCSD: Bias-correction and spatial disaggregation
DWS: Drinking water system
EC: Environment Canada
GCM: Global circulation model
GHG: Greenhouse gas
IPCC: Intergovernmental Panel on Climate Change
PCIC: Pacific Climate Impacts Consortium
RCP: Representative concentration pathway
WHO: World Health Organization
