# Supplementary material for: Projected local rain events due to climate change and the impacts on waterborne diseases in Vancouver, British Columbia, Canada
Source: Environ Health. 2019 Dec 30;18:116. doi: 10.1186/s12940-019-0550-y (PMC6937929; doi:10.1186/s12940-019-0550-y)
Supplement: Supplementary file 1 — Additional file 1: Figure S1. Map depicting the watersheds and supply areas for the Metro Vancouver Drinking Water System (Source: City of Surrey, BC, Canada, 2015). Figure S2. Comparison of weekly cumulative precipitation observed at Environment Canada weather station 698 and Pacific Climate Impacts Consortium (PCIC) interpolated precipitation for the grid containing the weather station (1997–2009). Figure S3. Time series of weekly cases of cryptosporidiosis and giardiasis, weekly cumulative precipitation, preceding dry days and average weekly turbidity from 1997 to 2009. [file 12940_2019_550_MOESM1_ESM.docx]

Figure S1. Map depicting the watersheds and supply areas for the Metro Vancouver Drinking Water System (Source: City of Surrey, BC, Canada, 2015).

Figure S2 Comparison of weekly cumulative precipitation observed at Environment Canada weather station 698 and Pacific Climate Impacts Consortium (PCIC) interpolated precipitation for the grid containing the weather station (1997- 2009).

Figure S3. Time series of weekly cases of cryptosporidiosis and giardiasis, weekly cumulative precipitation, preceding dry days and average weekly turbidity from 1997-2009.
